# CD163 as a novel target gene of STAT3 is a potential therapeutic target for gastric cancer

**DOI:** 10.18632/oncotarget.20244

**Published:** 2017-08-14

**Authors:** Zhenguo Cheng, Danhua Zhang, Baocheng Gong, Pengliang Wang, Funan Liu

**Affiliations:** ^1^ National Center for The International Research in Cell and Gene Therapy, Sino-British Research Centre for Molecular Oncology, School of Basic Medical Sciences, Academy of Medical Sciences, Zhengzhou University, Zhengzhou 450052, China; ^2^ Department of Surgical Oncology, The First Affiliated Hospital of China Medical University, Shenyang 110001, China; ^3^ Department of General Surgery, The First Affiliated Hospital of Zhengzhou University, Zhengzhou 450052, China

**Keywords:** CD163, gastric cancer, prognosis, STAT3, macrophages

## Abstract

CD163 is a member of the scavenger receptor cysteine-rich superfamily, and has been widely used to identify M2 type macrophage. However, the expression of CD163 in gastric cancer and its regulatory mechanism are still unclear. Here we show that CD163 is elevated in gastric cancer tissues. High expression of CD163 is a potential indicator to evaluate the status of tumor associated macrophages (TAMs), regulatory T cells (Tregs), myeloid-derived suppressor cells (MDSCs) and cancer associated fibroblasts (Cafs). Besides, more CD163 positive macrophages and CD163 expressing gastric cancer cells are associated with tumor invasion and poor prognosis. Knocking-down CD163 in cancer cells could inhibit tumor growth *in vivo*. We also find various immune molecules which are correlated with CD163 in gastric cancer tissues and cell lines have positive staining in the cancer cells of clinical sample. Finally, we confirm CD163 is a novel target gene of STAT3 (signal transducer and activator of transcription 3) in gastric cancer. Our data indicate that CD163 may be a potential poor prognostic marker and therapeutic target for gastric cancer.

## INTRODUCTION

CD163 (clusters of differentiation 163), also known as hemoglobin scavenger receptor or macrophage-associated antigen, is exclusively expressed in monocytes and macrophages [1]. This protein functions as an acute phase-regulated receptor involved in the clearance and endocytosis of hemoglobin-haptoglobin complexes by macrophages and defends against cytotoxic hemoglobin via autocrine and paracrine mechanism [2]. This also indicates CD163 may play a role in the uptake and recycling of iron, via endocytosis of hemoglobin-haptoglobin and subsequent breakdown of heme. In addition to the function of CD163 in the biology of normal physiology, high expression of CD163 is also involved in disease such as atherosclerosis, acute pancreatitis, diabetes and cancer [3–6].

Macrophages in tumor microenvironments play an important role in the suppression of adaptive immunity and promotion of angiogenesis and metastasis. In gastric cancer, CD204-positive macrophages is a significant risk factor [7]. CD163 has been used as a marker to evaluate macrophage infiltration, and its ectodomain (also called soluble CD163, sCD163) can be cleaved by ADAM17 (ADAM metallopeptidase domain 17) gene, which is closely related with tumor metastasis [8]. In ovarian cancer, high serum sCD163 levels are associated with a high grade tumor and poor prognosis [9]. In early stage melanoma, serum levels of sCD163 and macrophages infiltration at the tumor invasive front are independent predictors of survival [10]. Although study had found CD163 was increased in Helicobacter pylori infection [11], the expression of CD163 in gastric cancer is still unclear.

During the last decades, the development of immunotherapy based on tumor immune checkpoint blockade, greatly improve the survival of tumor patients [12]. Blocking antibody for immune checkpoints such as programmed death-ligand 1 (PD-L1), programmed cell death 1 (PDCD1) and cytotoxtic T-lymphocyte antigen 4 (CTLA4), have been used to treat several tumors including melanoma, renal cell carcinoma, lung cancer and gastric cancer [13, 14]. However, recent studies demonstrate macrophages can hijack anti-PDCD1 therapy by removing antibodies from T cells [15]. Some other studies find blocking tumor-infiltrating macrophages with CSF1R inhibitor benefits the therapeutic effect of PD1 and CTLA4 antagonists [11], indicating tumor-associated macrophage (TAM) is a promising tumor therapy target. Since CD163 is widely expressed in TAM, so understanding the expression and regulatory mechanism of CD163 in gastric cancer is important for gastric cancer therapy based on CD163.

In the recent study, we evaluate the expression of CD163 in gastric cancer tissues, and explore its function and regulatory mechanism in gastric cancer cell lines.

## RESULTS

### The mRNA expression of CD163 is elevated in gastric cancer (GC)

**Figure 1:**
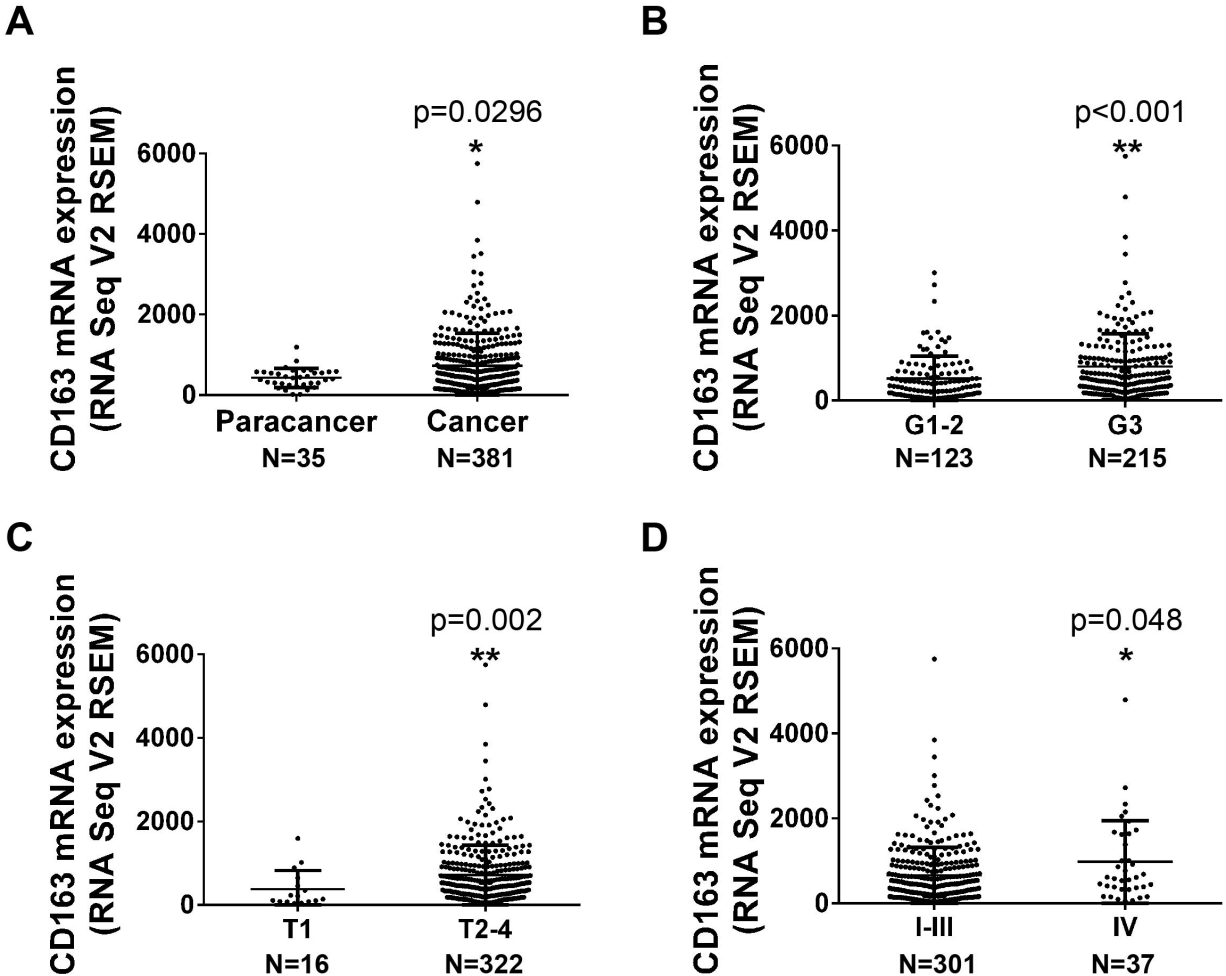
The mRNA expression of CD163 in gastric cancer tissues from TCGA database **(A)**, The expression of CD163 in paracancer (35) and cancer tissues (381). **(B-D)**, The expression of CD163 in different histologic grade (B), depth of invasion (C) and TNM stage (D) (338 of 381 cancer tissues had clinical data).

The mRNA expression data of 416 gastric tissues (35 paracancer tissues and 381 tumor tissues) was downloaded from TCGA (The Cancer Genome Atlas) database and normalized with TCGA Assembler and R software following the previous study [7]. Figure 1A showed that CD163 mRNA is significantly increased in caner tissues compared with paracancer tissues. Subsequently, the relationship between CD163 expression and clinicopathologic characters was assessed. Results displayed poor histologic grade patients possessed a high CD163 level (Figure 1B, p<0.001). Besides, CD163 expression was also associated with tumor invasion (Figure 1C, p<0.01). However, there were no significant correlation was found in different age, gender, lymph node metastasis and distant metastasis (p>0.05) (Table 1). Although CD163 expression in TNM **IV** is higher than it in TNM I-III, it seemed that change was mainly caused by tumor invasion status (Figure 1D) (p=0.048).

**Table 1:** Correlation between CD163 mRNA expression and clinicopathological characteristics in gastric cancer

*Characters*	*No.*	*Log2(RSEM)(mean±SD)*^a^	*p* value^b^
Age(yrs)			
<60	111	8.79±1.44	0.988
≥60	227	8.79±1.47	
Gender			
Male	215	8.76±1.46	0.631
Female	123	8.84±1.47	
Histologic grade			
Well & Moderate	123	8.34±1.46	0.000013 ^**^
Poor	215	9.05±1.40	
Depth of invasion (pT)			
T_1_	16	7.67±1.67	0.002 ^**^
T_2_, T_3_, T_4_	322	8.85±1.43	
Lymph node status (pN)			
N_0_	106	8.64±1.49	0.201
N_1_, N_2_, N_3_	232	8.86±1.44	
Tumor metastasis (pM)			
M_0_	316	8.76±1.45	0.173
M_1_	22	9.20±1.60	
Pathological stage (TNM)			
I,II,III	301	8.73±1.44	0.048 ^*^
IV	37	9.24±1.56	

^a^ Mean of relative expression by log2(RSEM value), with mean±SD.

^b^ One-Way ANOVA was used for analysis, ^*^p<0.05, ^**^p<0.01.

### CD163 is an index for infiltration of tumor associated macrophages, Tregs, MDSCs and Cafs

**Figure 2:**
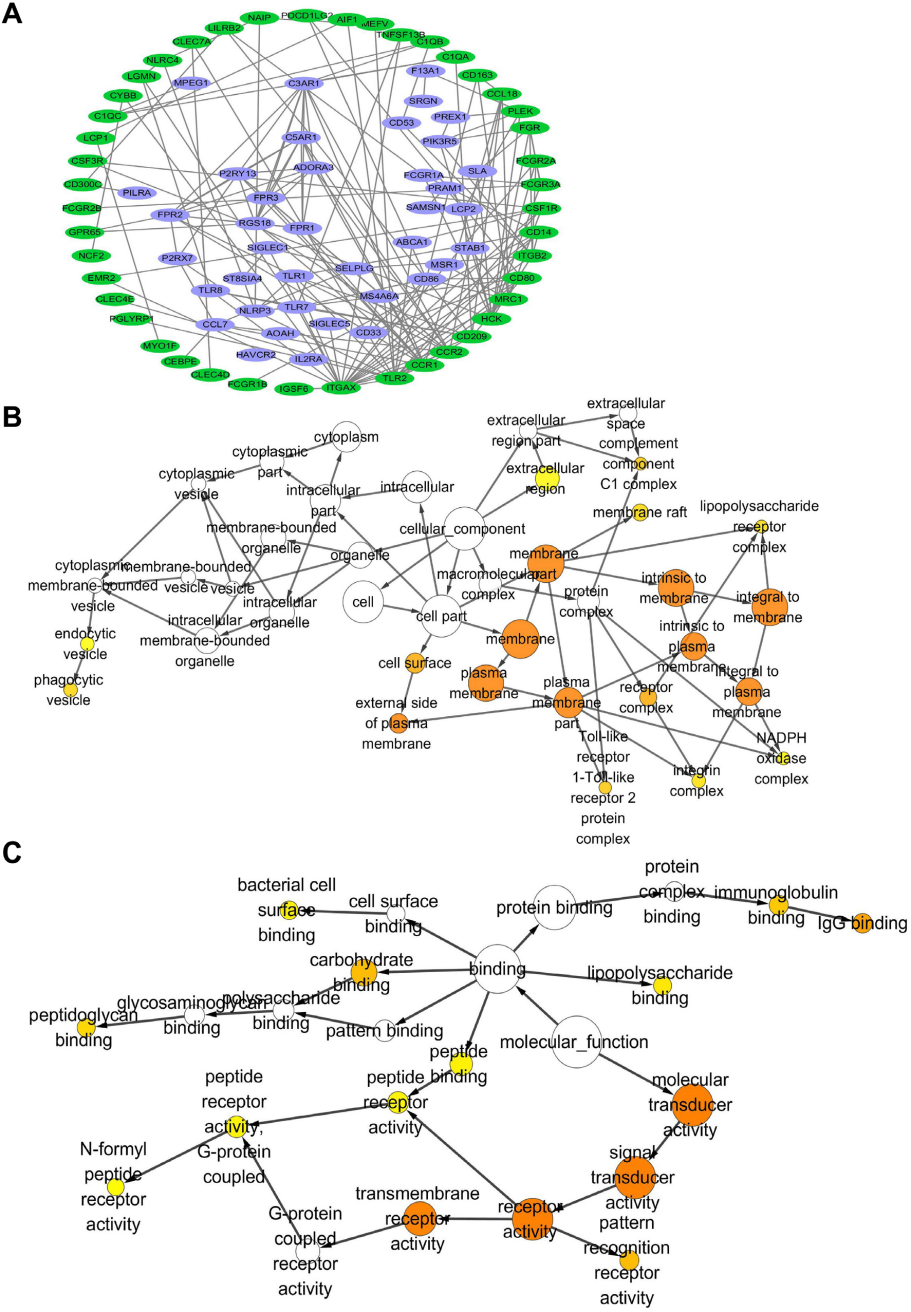
Protein interaction and gene ontology consortium analysis between genes that co-expressing with CD163 in cancer tissues **(A)**, Protein interaction network between CD163 co-expressing genes (R>0.5) were analyzed with String and Cytoscape, genes that involved in immune responses are marked with green color. Cellular component **(B)** and molecular function **(C)** enrichment analysis of genes in protein interaction network are performed with Cytoscape, Orange color represents p<0.01.

To further understand the clinical significance of CD163, protein interaction network among co-expressing genes which had high consistency (r >0.5) in GC tissues were analyzed with String software. As shown in Supplementary Table 1, all top 10 functional pathways were associated with immune response. Figure 2A showed the core interaction network of these co-expressing genes, and immune related genes were marked with green. We also found that CD163 could directly interact with CCL18 and CD86 which were associated with T-lymphocyte activation, indicating CD163 may be involved in cancer immunosurveillance. Additionally, cellular component enrichment analysis were performed with Cytoscape software. Figure 2B showed that the co-expressing genes were mainly enriched in cell membrane and extracellular components. Besides, these genes were involved in several pathways including TLR2, integrin, NADPH, lipopolysaccharide, complement and endocytosis hinting they were potential therapeutic targets or diagnostic serum markers. To better understand potential function of CD163 molecular function enrichment analysis was also performed. As shown in Figure 2C, CD163 correlated genes mainly participated in receptor recognition, immunoglobulin binding and peptidoglycan binding which were involved in the process of immunomodulation.

**Figure 3:**
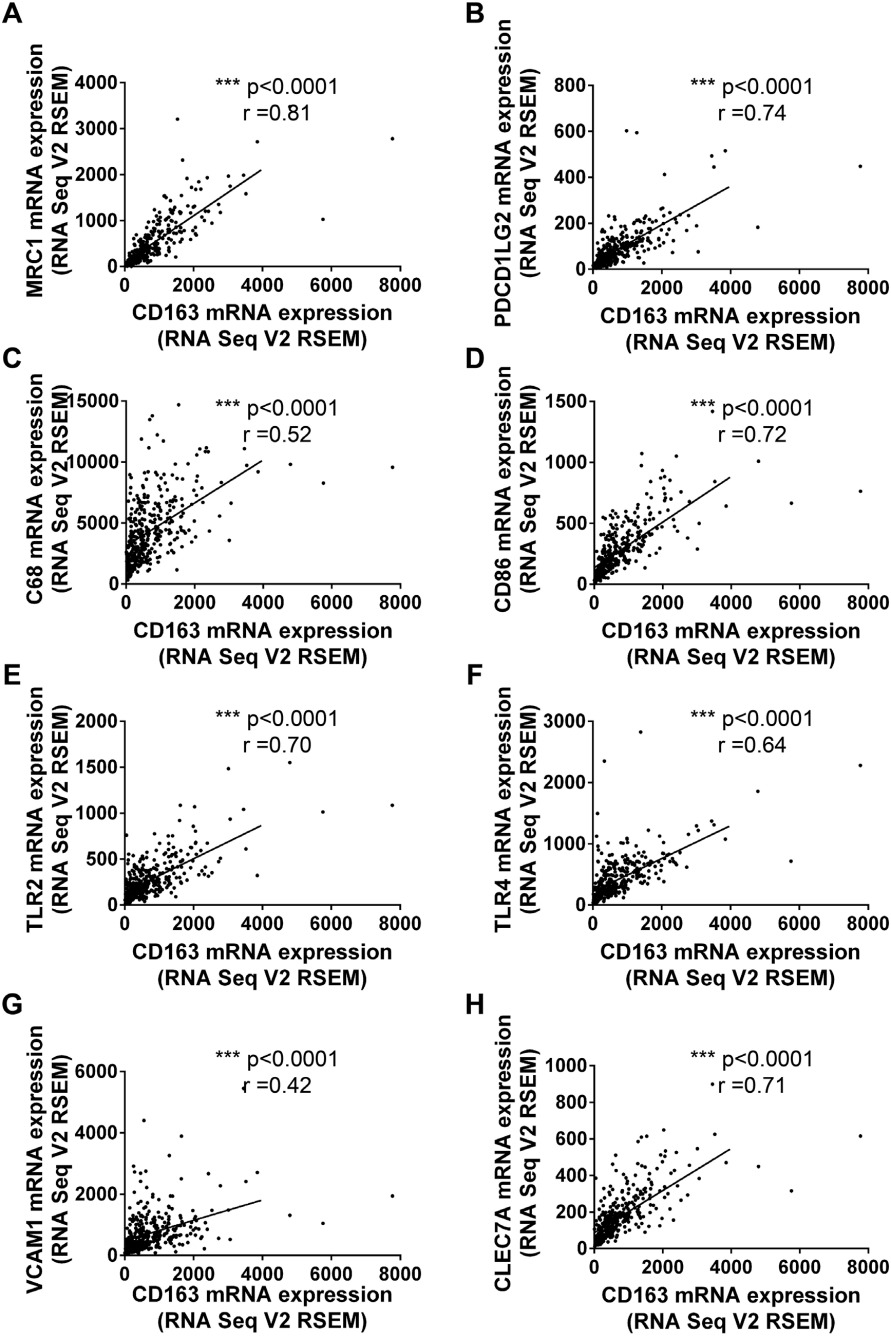
The correlationship between CD163 and markers of macrophages M2 markers, MRC1 **(A)** and PDCD1LG2 **(B)**; M1 markers, CD68 **(C)**, CD86 **(D)**, TLR2 **(E)** and TLR4 **(F)**; TAM markers, VCAM1 **(G)** and CLEC7A **(H)** are analyzed with R software.

**Figure 4:**
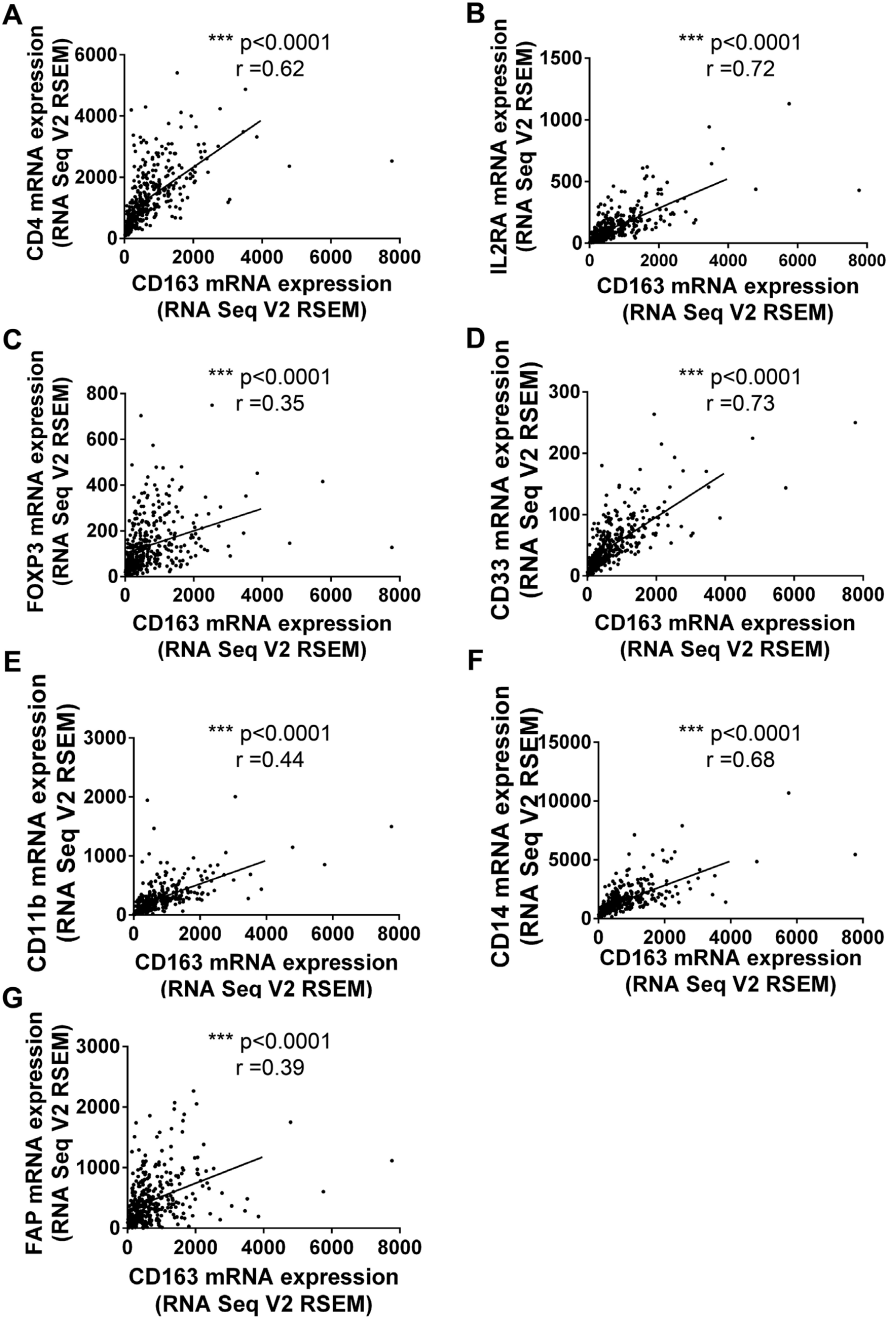
The correlationship between CD163 and markers of Tregs, MDSCs and CAFs Tregs markers, CD4 **(A)**, IL2RA **(B)** and FOXP3 **(C)**; MDSCs markers, CD33 **(D)**, CD11b **(E)**, CD14 **(F)**; CAFs markers, FAP are analyzed with R software.

Numerous studies had confirmed tumor associated macrophages (TAMs), Tregs, MDSCs and Cafs in tumor microenvironment stroma were crucial risk factor for tumor immune escape and poor prognosis [16, 17]. So we tried to examine whether CD163 could be used as a potential indicator to evaluate the status of tumor microenvironment. Firstly, we assessed the correlation of CD163 with markers for macrophages. As expected, the correlation coefficients of CD163 with MRC1 (Figure 3A) and PDCD1LG2 (Figure 3B) were 0.81 and 0.74, which had been widely used to mark type 2 macrophages (M2). Then, markers of type 1 macrophages (M1) including CD68, CD86, TLR2 and TLR4 were examined. As shown in Figure 3C-3F, close positive correlation was found in these indicators with CD163. Our data also confirmed CD163 was associated with VCAM1 (r=0.42) and CLECA7 (r=0.71) which had been used to identify tumor associated macrophages recently (Figure 3G and 3H). Secondly, we assessed the correlation between CD163 expression with Tregs status by analyzing CD4, IL2RA and FOXP3. Results showed the correlation coefficients of CD163 with CD4, IL2RA and FOXP3 were up to 0.62, 0.72 and 0.35 (Figure 4A-4C). Similar results were observed in the analysis data of CD33, CD11b and CD14 which were markers for MDSCs (Figure 4D-4F). Finally, correlation between CD163 and Cafs status was evaluated by using fibroblast activation protein alpha (FAP) which had been confirmed elevated in Cafs. As shown in Figure 4G, the expression of CD163 was also associated with FAP level.

### High expression of CD163 in both macrophages and cancer cells are associated with poor prognosis

**Figure 5:**
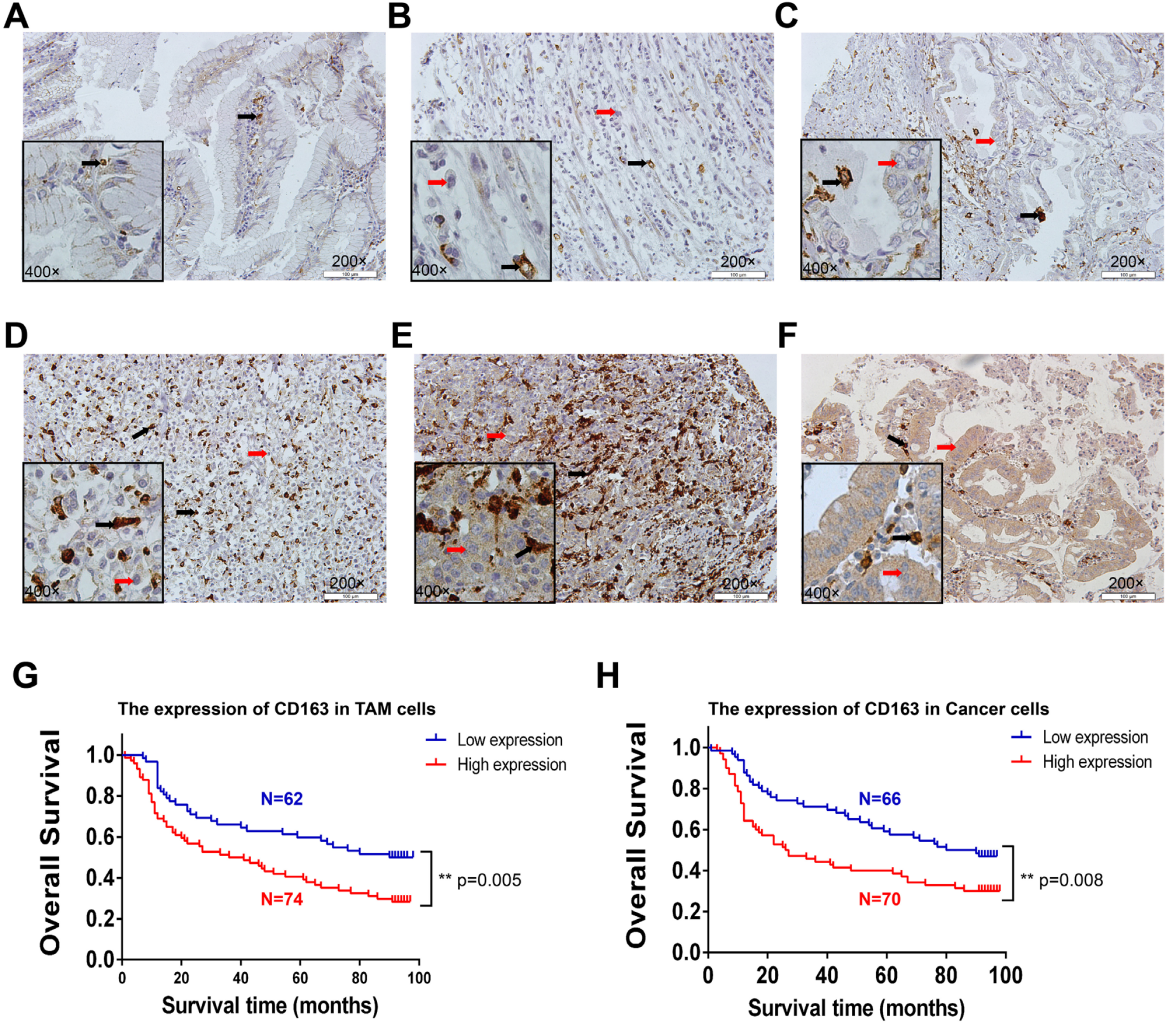
The expression and clinical significance of CD163 in macrophages and cancer cells The staining of CD163 in para-cancer **(A)** and cancer tissues **(B-F)**. B represents macrophages (grade 1) and cancer cells (negative); C represents macrophages (grade 2) and cancer cells (negative or low); D represents macrophages (grade 3) and cancer cells (negative or low); E represents macrophages (grade 4) and cancer cells (moderate); F represents macrophages (grade 2) and cancer cells (high). Red arrow is cancer cell, black arrow is macrophage. The survival curve of CD163-positive macrophages **(G)** and cancer cells **(H)** in 136 tumor tissues are analyzed with SPSS software (3 of 139 patients that had no survival data were removed).

Considering tumor tissues were mixture of cancer cells and tumor stroma, we next examined the protein expression of CD163 in 139 tumor tissues and 10 para-cancer tissues by immunohistochemestry assay. Consistent with previous results [18], a large numbers of CD163 positive macrophages could be found in tumor stroma. Strikingly, CD163 staining also could be observed in some cancer cells. Different staining intensity of CD163 in para-cancer and cancer tissues were displayed in Figure 5A-5F. Through analyzing clinical and pathological characters, we found CD163 positive macrophages counts were significantly higher in patients with poor degree of differentiation and a deeper invasion (Table 2). This result was consistent with the mRNA change in TCGA gastric cancer tissues, suggesting macrophages in tumor microenvironment played an important role in the differentiation and invasion of cancer cells. It was interesting that elevated expression of CD163 in cancer cells was only associated with the depth of invasion, but not with histologic grade (Table 3). Additionally, survival analysis of patients were performed. Similar to the reported results in other cancers, our results also confirmed patients with more macrophages infiltration had a shorter survival time (Figure 5G). Interestingly, the survival time of patients with high expression of CD163 in cancer cells was significantly reduced too (Figure 5H). Further Multivariate analysis demonstrated that CD163 positive macrophages (Supplementary Table 2) and cancer cells (Supplementary Table 3) were potential risk factors for patients’ survival.

**Table 2:** Correlation between the CD163 expression in TAM cells and clinicopathological characteristics in gastric cancer

*Characters*	*No.*	*Expression of CD163*		*p* value^a^
		Low*N=64*	High*N=75*	
Age(yrs)				
<60	70	35 (50.0%)	35 (50.0%)	0.396
≥60	69	29 (42.0%)	40 (58.0%)	
Gender				
Male	35	18 (51.4%)	17 (48.6%)	0.557
Female	104	46 (44.2%)	58 (55.8%)	
Histologic grade				
Well & Moderate	60	36 (60.0%)	24 (40.0%)	0.006 ^**^
Poor	79	28 (35.4%)	51 (64.6%)	
Depth of invasion (pT)				
T_1_ - T_2_	35	22 (62.9%)	13 (37.1%)	0.03 ^*^
T_3_ - T_4_	104	42 (40.4%)	62 (59.6%)	
Lymph node status (pN)				
N_0_ – N_1_	90	43 (47.8%)	47 (52.2%)	0.598
N_2_ – N_3_	49	21 (42.9%)	28 (57.1%)	
Pathological stage (TNM)				
I-II	86	44 (51.2%)	42 (48.8%)	0.161
III-IV	53	20 (37.7%)	33 (62.3%)	

^a^ Chi-Square test was used for analysis, ^*^p<0.05, ^**^p<0.01.

**Table 3:** Correlation between the CD163 expression in cancer cells and clinicopathological characteristics in gastric cancer

*Characters*	*No.*	*Expression of CD163*		*p* value^a^
		Low *N=68*	High *N=71*	
Age(yrs)				
<60	70	37 (52.9%)	33 (47.1%)	0.398
≥60	69	31 (44.9%)	38 (55.1%)	
Gender				
Male	35	14 (40.0%)	21 60.0%)	0.246
Female	104	54 (51.9%)	50 (48.1%)	
Histologic grade				
Well & Moderate	60	34 (56.7%)	26 (43.3%)	0.126
Poor	79	34 (43.0%)	45 (57.0%)	
Depth of invasion (pT)				
T_1_ - T_2_	35	23 (65.7%)	12 (34.3%)	0.031 ^*^
T_3_ - T_4_	104	45 (43.3%)	59 (56.7%)	
Lymph node status (pN)				
N_0_ – N_1_	90	47 (52.2%)	43 (47.8%)	0.598
N_2_ – N_3_	49	21 (42.9%)	28 (57.1%)	
Pathological stage (TNM)				
I-II	86	46 (53.5%)	40 (46.5%)	0.221
III-IV	53	22 (41.5%)	31 (58.5%)	

^a^ Chi-Square test was used for analysis, ^*^p<0.05, ^**^p<0.01.

**Figure 6:**
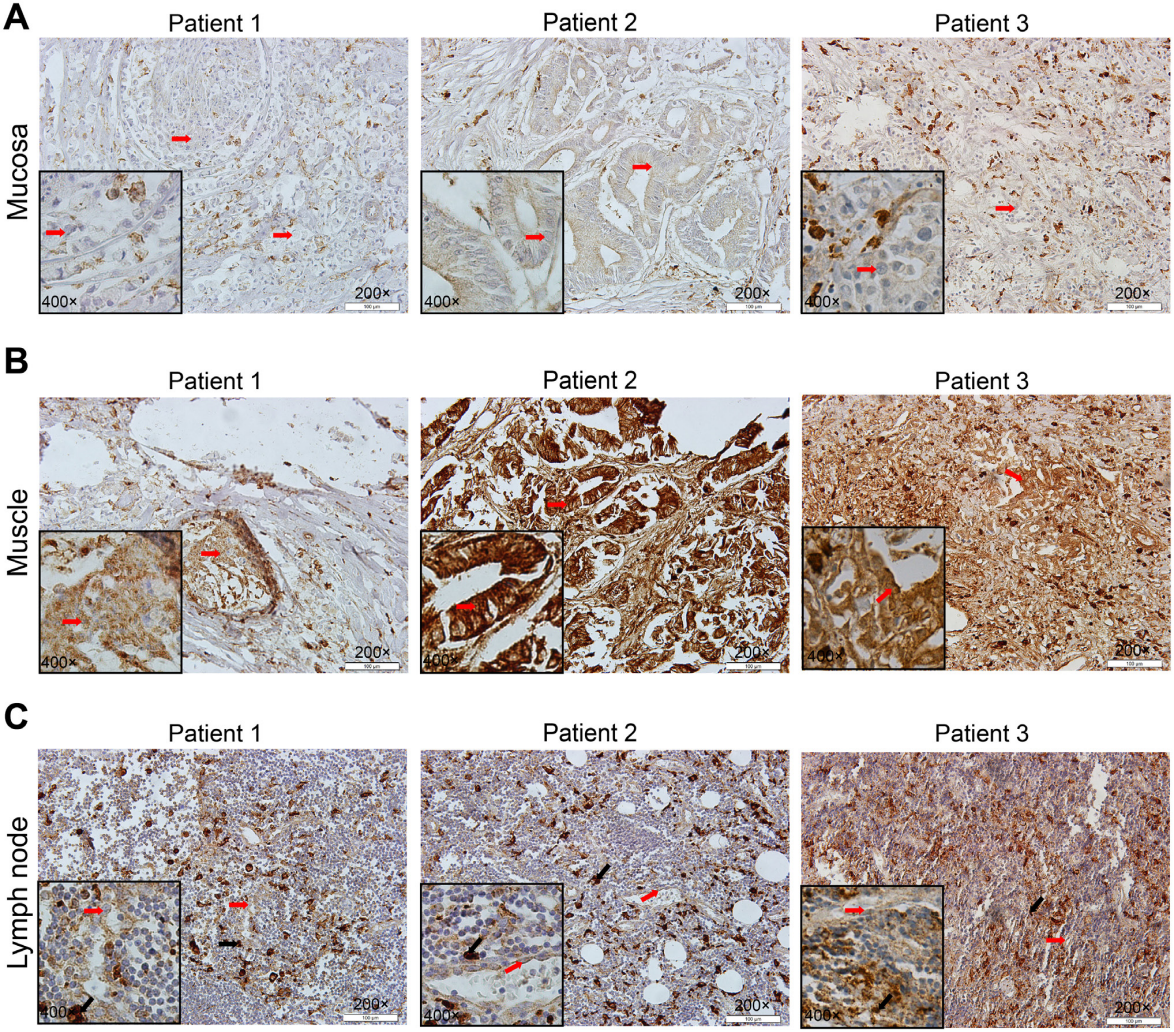
Different expression of CD163 in gastric mucosa, muscle layer and metastatic lymph node Cancer cells in muscle layer **(B)** have a higher CD163 expression than in gastric mucosa **(A)**. There are massive CD163 positive macrophages in metastatic lymph node **(C)**. Patient 1, signet ring cell carcinoma; patient 2, gastric tubular adenocarcinoma; patient 3, poorly differentiated glandular carcinoma. Red arrow is cancer cell and black arrow is macrophage.

To explore the potential function of CD163 in gastric cancer cells, we compared the expression of CD163 in cancer cells located in gastric mucosa with that invaded into muscle layer from same patients. As shown in Figure 6, CD163 was expressed in some tumor cells at the mucosa layer (Figure 6A). While when tumor cells broken lamina propria of the mucosa, CD163 significantly increased (Figure 6B and Supplementary Figure 1A). As study had reported tumor cells play an important role in the formation and transformation of macrophages [19], so we also examined CD163 in the metastatic lymph node. Strikingly, there were a large numbers of infiltrated CD163 positive macrophages in these metastatic lymph nodes, indicating CD163 positive macrophages might be the key cause of tumor immune evasion in lymph nodes. While, the expression of CD163 in cancer cells of lymph node had no significant alteration compared with its expression in primary tumor cells, suggesting CD163 in cancer cells may not be a crucial risk for lymphatic metastasis (Figure 6C and Supplementary Figure 1A).

### Knocking-down of CD163 in cancer cells inhibits tumor growth *in vivo*

**Figure 7:**
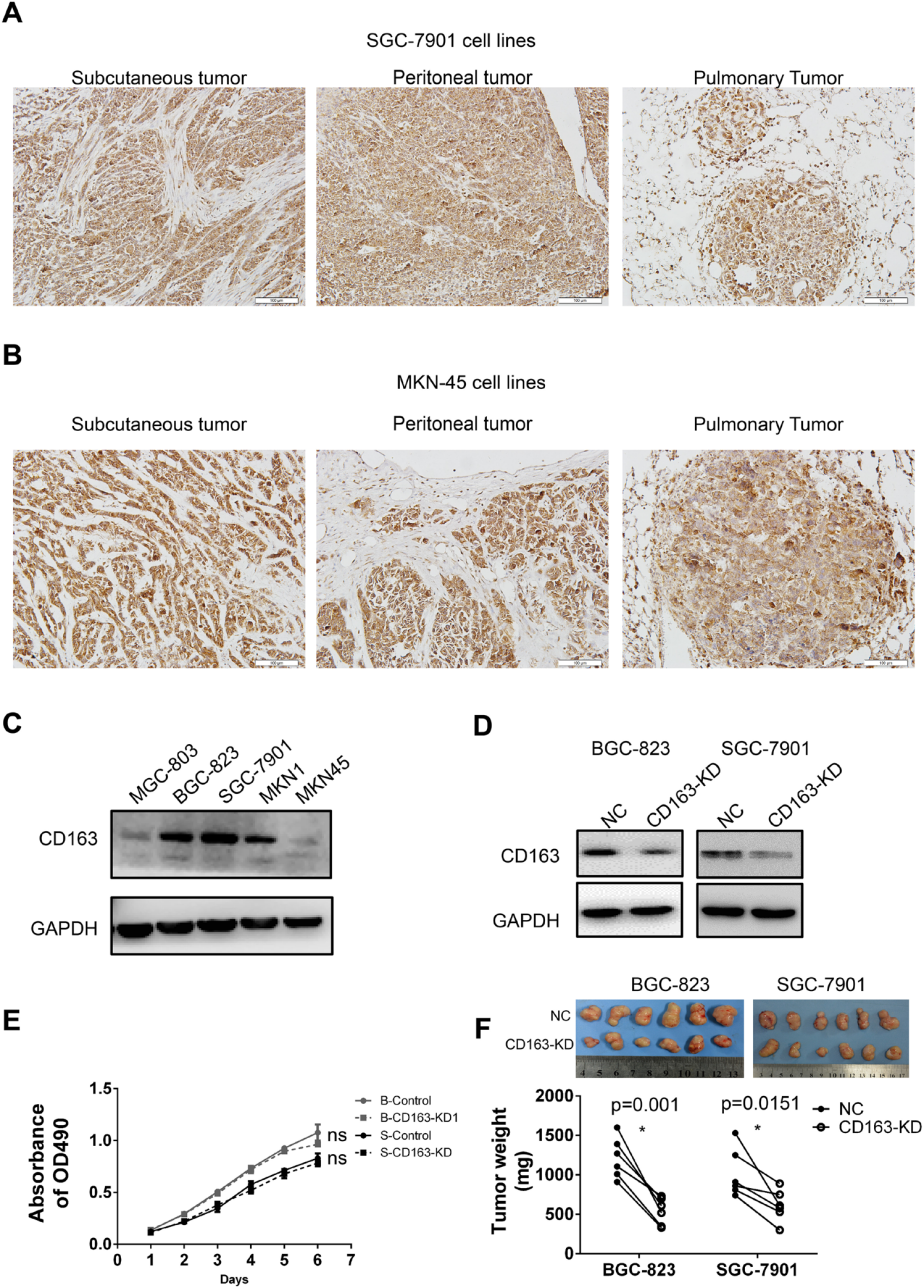
The expression and function of CD163 in gastric cancer cells CD163 expression in different tissues xenografts (subcutaneous, peritoneal and pulmonary cancer) from SGC-7901 **(A)** and MKN-45 **(B)** transplanted nude mice are examined by immunohistochemistry assay (200×). The expression of CD163 in 5 gastric cancer cell lines **(C)** and CD163 knocking-down stable cell lines **(D)** are detected by Western blot. The effect of CD163 on cell growth *in vitro* and in *in vivo* are evaluated by MTT **(E)** and tumor-bearing nude mice respectively **(F)**. ns, no significant difference; “B” and “S” in panel E represent “BGC-823” and “SGC-7901” respectively.

In order to examine the role of CD163 in tumor cells, we firstly detected its expression in several gastric cancer cell lines. As shown in Figure 7C and Supplementary Figure 1C, CD163 was expressed in all five gastric cancer cells. The high level in BGC-823 (poor differentiation), SGC-7901 (moderate differentiation), MKN1 (high differentiation) and low level in MGC-803 (poor differentiation), MKN-45 (poor differentiation) revealed CD163 had no correlation with cell differentiation, which was consistent with the above results.

To explore whether CD163 is associated with tumor metastasis, the expression of CD163 in xenografts from different tissues of nude mice were evaluated. Results revealed that CD163 was strongly expressed in all these xenografts from SGC-7901 (Figure 7A) and MKN45 (Figure 7B). But cancer cells in subcutaneous, peritoneal and pulmonary had no significantly difference (Supplementary Figure 1B), suggesting CD163 in cancer cells had weak influence on metastasis in nude mice. Although CD163 expression in MNN-45 was markedly weak *in vitro* compared with SGC-7901, this difference was not obviously *in vivo*, hinting tumor microenvironment might be involved in the regulation of CD163.

Finally, we generated CD163 knocking-down cell lines with lentivirus-coated shRNA, and the silencing efficiency was verified (Figure 7D and Supplementary Figure 1D). MTT assay was performed to assess the function of CD163 on cell growth *in vitro*. Unexpectedly, no significant difference was seen between negative control (NC) and CD163 knocking-down groups (CD163-KD) (Figure 7E). However, when these cells were injected into the bilateral axillary of nude mice, xenografts from CD163-KD were smaller than that from NC group (Figure 7F). This result implied the effect of CD163 on cancer growth was related with tumor microenvironment.

### CD163 is co-expressing with immune related molecules in gastric cancer cells

**Figure 8:**
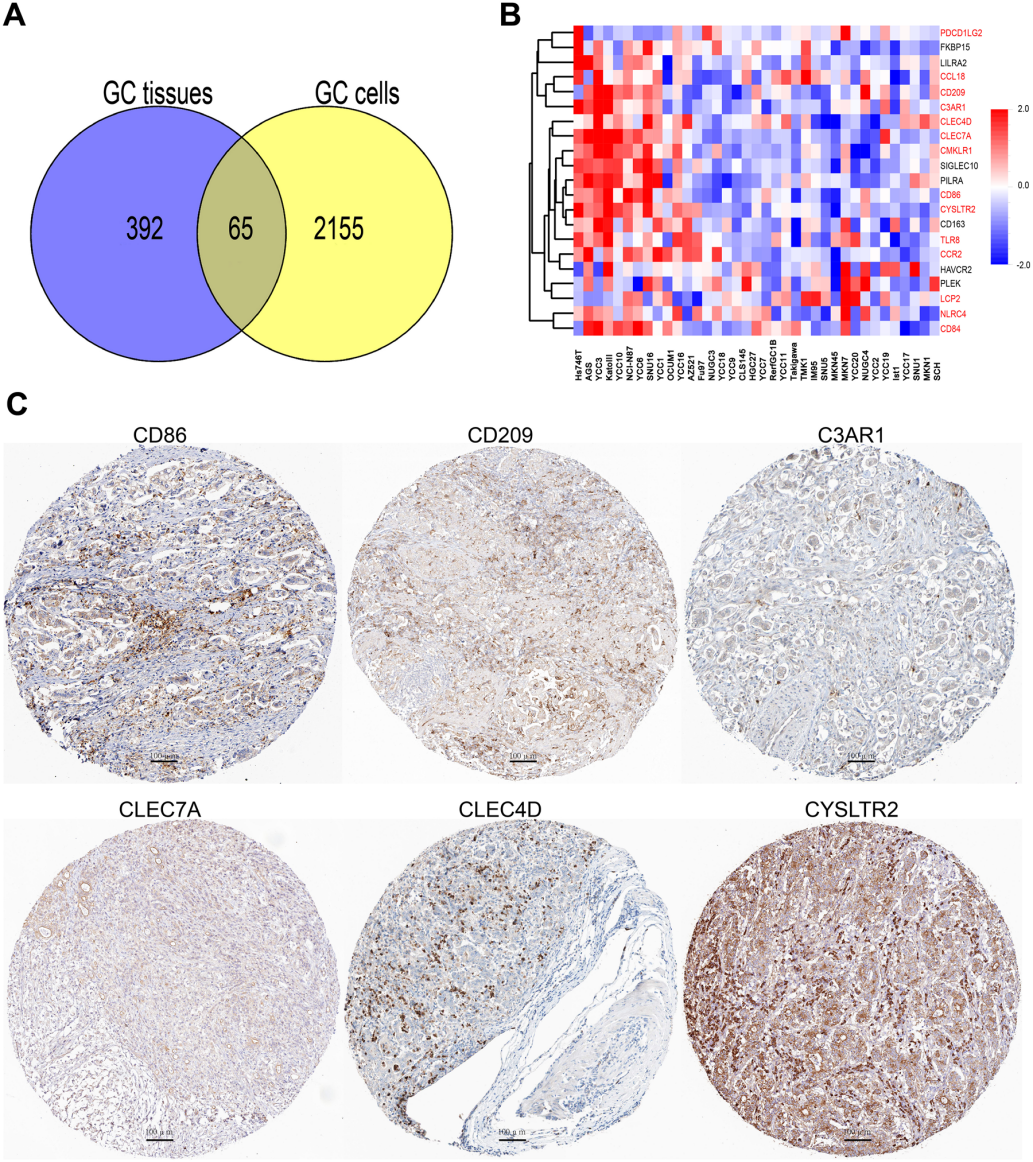
CD163 is co-expressing with various immune related genes in cancer cells **(A)**, Overlap analysis of genes that co-expressing with CD163 in 37 cell lines and 381 gastric cancer tissues. **(B)**, Top 20 overlapped genes in 37 cancer cell lines are enriched and clustered with R software. **(C)**, Protein expression of CD86, CD209, C3AR1, CLEC7A, CLEC4D and CYSLTR2 are evaluated in protein atlas database (scanning at 200× magnification).

As mentioned above that stroma cells in cancer tissues may interfere the results of CD163 in cancer cells. In an effort to examine the potential function of CD163 in gastric cancer cells, genes which were co-expressing with CD163 in 37 gastric cancer cell lines and 381 gastric cancer tissues were analyzed (correlation coefficient >3.5). Results showed there were 65 overlapped genes which had positive correlation with CD163 (Figure 8A). Then the top 20 overlapped genes in cancer cell lines were enriched and clustered with R software. As shown in Figure 8B, many immune related molecules such as CCL18, CD209, C3AR1, CLEC4D, CLEC7A, CMKLR1, CD86, CYSLTR2, TLR8 and CCR2 were consistent with CD163, suggesting CD163 expressed in cancer cells may also be involved in tumor immune evasion. Subsequently, we blasted these immune related proteins in Protein Atlas database (www.proteinatlas.org) [20], and found that proteins including CD86, CD209, C3AR1, CLEC4D, CLEC7A and CYSLTR2 have positive staining in cancers cells (Figure 8C). This finding was consistent with several other studies which had confirmed that genes such as CD209 [21], C3AR1 [22], CLEC7A [23] and CYSLTR2 [23] had differentially expression in cancer cells. We also evaluated the correlation between CD163 expression with two key immune checkpoints PD-L1 and CTLA4 in gastric cancer cell lines. As shown in Supplementary Figure 1E and 1F, both these two checkpoints were positively correlated with CD163, indicating CD163 might be a novel immunomodulatory protein.

### CD163 is a downstream gene of STAT3

**Figure 9:**
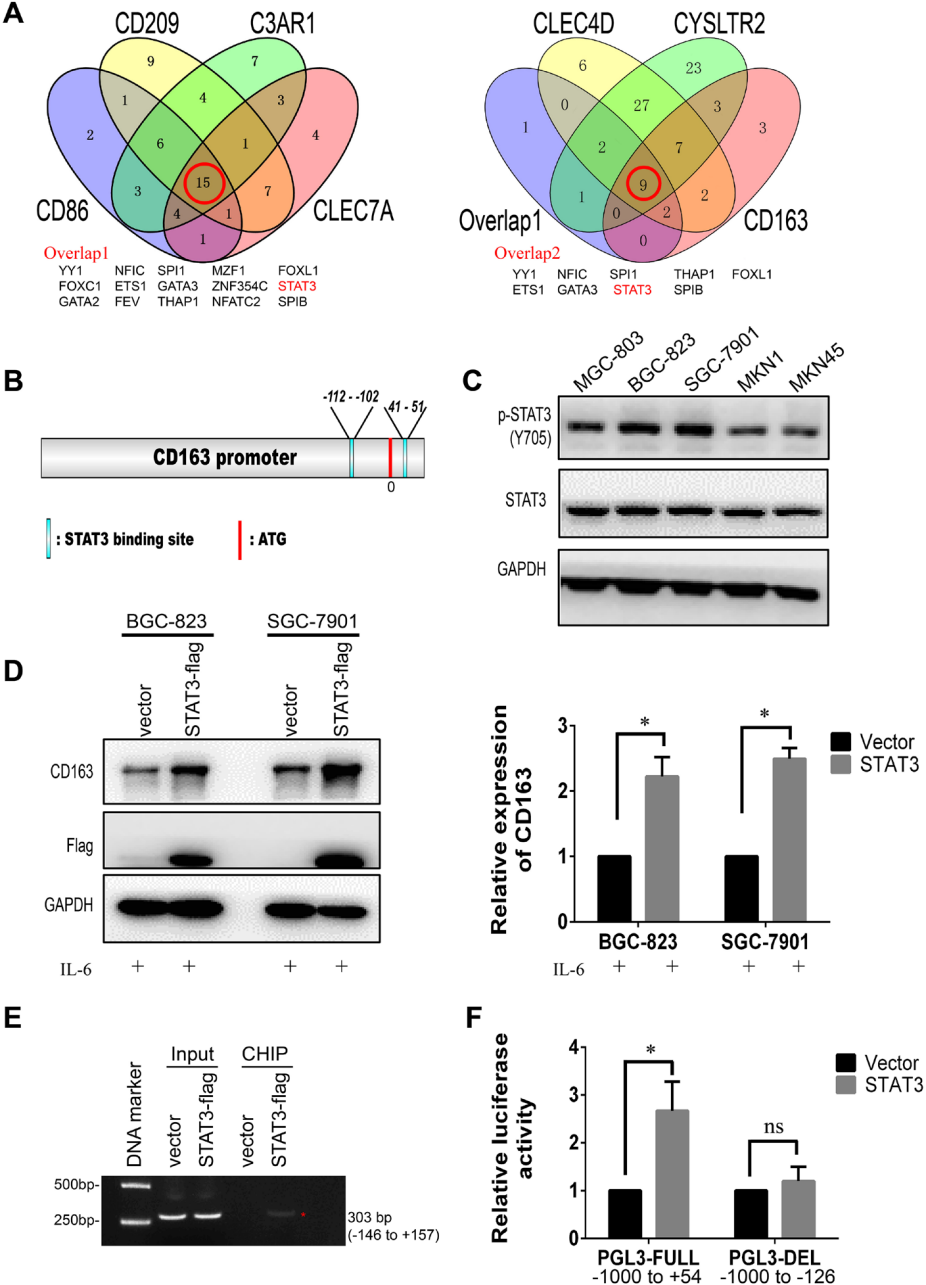
CD163 is a novel target gene of STAT3 **(A)**, Overlapped transcriptional factors of CD86, CD209, C3AR1, CLEC7A, CLEC4D, CYSLTR2 and CD163 are identified by Venny software. **(B)**, Potential STAT3 binding sites on the promoter of CD163 is predicted by JASPAR. **(C)**, The expression of STAT3 and activated STAT3 (pY705 STAT3) in gastric cancer cell lines are detected with Western blot. **(D)**, Relative expression of CD163 after overexpressing STAT3 is validated by Western blot assay. **(E)**, STAT3- DNA complex in SGC-7901 is isolated by Chromatin immunoprecipitation, and potential binding region is identified by PCR. **(F)**, The effect of STAT3 on promoter activity of full length type or deletion type is evaluated by luciferase reporter assay.

To explore the potential transcriptional regulator of CD163, the promoter of CD163, CD86, CD209, C3AR1, CLEC4D, CLEC7A and CYSLTR2 were predicted by JASPAR, then overlap transcriptional factors were analyzed by Venny software. Results demonstrated there were 9 potential regulators shared by these genes (Figure 9A). Considering STAT3 was an important node that mediated cellular responses to cytokines and acted as crucial regulator of PD-L1 and CD86 in cancers [23, 24], we focused our attention on STAT3. As shown in Figure 9B, potential STAT3 binding sites on the promoter of CD163 were analyzed by JASPAR. To verify this hypothesis, we first examined the expression of STAT3 and activated STAT3 (p-Y705) in 5 gastric cancer cell lines. Results showed total STAT3 had no significant difference between them, but activated STAT3 was high expressed in BGC-823 and SGC-7901 cells (Figure 9C). This change indicated hyperactivation of STAT3 may be the main cause of high CD163 expression. Subsequently, we hoisted STAT3 expression in gastric cancer cells by transfecting STAT3 plasmid and activated it by stimulating with IL-6. As expected, overexpressing STAT3 in cancer cells was able to elevate the expression of CD163 (Figure 9D). To further validate the regulation of CD163 by STAT3, chromatin immunoprecipitation and luciferase reporter assays were performed. As shown in Figure 9E, STAT3 could interact with the potential binding motif of CD163 promoter. From Figure 9F we could find overexpressing STAT3 was able to elevate relative luciferase activity of wild type CD163 promoter, while had no significant effect on deletion promoter. Totally, all these findings confirmed that STAT3 was an important regulator of CD163 in gastric cancer cells.

## DISCUSSION

CD163 as a marker of macrophages, has been used to evaluate the status of tumor macrophages. Generally, tumor macrophages are categorized into two subsets, M1 macrophages and M2 macrophages [25]. Phenotypically, M1 macrophages often have enhanced expression of CD68, CD86, CD169, TLR2 and TLR4, whereas, M2 macrophages possess high level of CD163, CD206 (MRC1), PDCD1LG2 and CD204 (MSR1). In a tumor environment, the majority of tumor associated macrophages (TAMs) are considered to be the M2 macrophages. M2 macrophages not only inhibit tumor apoptosis and promote cell proliferation by activating NK-κB [26], but participated in tumor metastasis by releasing diverse pro-angiogenic factors such as VEGF, FGF2 and TNFα [27]. It is known that M2 macrophages can promote an immunosuppressive environment by secreting various immunomodulatory molecules such as IL-10, CCl2, CSF-1 and TGF-β [28]. Recently, high protein expression of CD163 is observed in oral squamous carcinoma [29], meningioma [30], bladder cancer [31], breast cancer [32]. Elevated soluble CD163 in serum is a potential biomarker for the diagnosis of myeloma and ovarian cancer [9, 33]. In stomach, elevated CD163 is found in Helicobacter pylori infection [11]. Here, we find that the mRNA expression of CD163 is elevated in GC tissues, and confirm patients with more CD163 positive macrophages have a poor prognosis. Correlating analysis demonstrates increasing CD163 has positive correlation with others TAMs markes including MRC1, PDCD1LG2, VCAM1, CLEC7A. Although M1 macrophages has anti-tumor function, our data shows markers including CD68, CD86, TLR2 and TLR4 are correlated with CD163 too. This phenomenon is not incomprehensible, because M1 macrophages could be induced into M2 macrophages by some tumor derived cytokines such as CSF-1, IL10 and TGF-β [34]. In this paper, the massive CD163 positive macrophages which are observed in the metastatic lymph nodes also favors this hypothesis. Data reported here also confirm elevated CD163 is correlated with tumor differentiation and invasion depth. This finding is consistent with another study in which researchers demonstrate that infiltrating TAM can increase epithelial mesenchymal transition (EMT) via activating AKT/mTOR pathway [35].

During the last decades, cancer immunosurveillance which becomes a novel tumor therapeutic target attracts more and more scientists’ attention. Many cell types including TAMs, Tregs, MDSCs and Cafs accumulate in tumor environment and develop an adaptive immune niche which protects tumors from immunosurveillance. These suppressive cells markedly restrict the function of NKs (natural killer cells), DCs (dendritic cells) and cytotoxic T cells by secreting diverse cytokine or expressing suppressive immune signal such as PD-L1 and CTLA4 [36]. Studies have found molecules secreted by TAMs such as IL-10, IL-6, CSF-1 inhibit dendritic cells maturation [37]. Moreover, TAM derived TGF-β, PGE2, IL-10, and cytokines such as CCL2, CCL17 and CCL18 recruit abundant Tregs to tumor cells [38]. Additionally, interaction between TAMs and Cafs can induce recruitment and activation each other [39]. Here, our data confirms CD163 in gastric cancer has a positive correlation with Tregs (CD4, CD25 and FOXP3), MDSCs (CD33, CD11b and CD14) and Cafs (FAP), indicating CD163 level is an potential index to monitor the status of tumor associated macrophages, Tregs, MDSCs and Cafs in gastric cancer.

Although CD163 has been widely used as a marker of M2 macrophages, the function of it is still confined to the endocytic receptor of hemoglobin/haptogolbin complex. Some researchers speculate CD163 may orchestrate tissues repair by denting the pro-inflammatory cytokine TWEAK which is a regulator of tissues injury [40]. In addition, TWEAK as a protective role in gastrointestinal tumors also gets confirmed [41, 42]. Our protein interaction analysis reveals CD163 together with various immune related genes could form a complex network. Moreover, most of these interacting proteins are membrane proteins which participate in receptor recognition and signal transduction. Subsequently, we find CD163 is high expression in some gastric cancer cells especially in muscle layer invaded cells. High CD163 level in cancer cells is a potential marker of poor prognosis. Further experiments demonstrate knocking-down CD163 in gastric cancer cell is able to inhibit tumor growth *in vivo*, but have no effect on cell growth *in vitro*, indicating the function of CD163 in cancer cells may depend on the stimulation of extracellular signals. Overlap analysis with genes that co-expressing in 381 gastric cancer tissues and 37 gastric cells demonstrates that various immune related genes such as CCL18, TLR8, C3AR1, CYSLTR2 and CD86 are positively correlated with CD163. Furthermore, results from Protein Atlas database validate immune molecules including CD86, CD209, C3AR1, CLEC4D, CLEC7A and CYSLTR2 are positive in gastric cancer cells. We also verify another two immune checkpoints PD-L1 and CTLA4 have positively correlation with CD163 in cancer cells. All these findings indicate CD163 in cancer cells promotes tumor progress by participate immunomodulation.

STAT3 is a member of STAT family that regulates gene transcription by relaying signals from plasm membrane to the nucleus [43]. It is well known that STAT3 and its downstream genes not only promote cancer growth, survival, angiogenesis and metastasis, but also interfere with cellular apoptosis and anti-tumor immune responses [44]. In stomach, hyperactivation of STAT3 directly promote gastric carcinogenesis by increasing the epithelial expression of Toll-like receptor 2 (TLR2) [45]. Moreover, IL-6/STAT3 signaling promotes gastric cancer invasion and migration by increasing CD44 variant 6 [46]. Here, we identity CD163 together with its co-expressing genes including CD86, CD209, C3AR1, CLEC4D, CLEC7A and CYSLTR2 possess potential STAT3 binding motif by bioinformatics analysis. Subsequently, we confirmed overexpression of STAT3 can enhance the CD163 level in gastric cancer cells. Through CHIP and luciferase reporter assays, we further confirm CD163 is a novel downstream gene of STAT3.

In summary, our data demonstrate that CD163 is a novel predictor to evaluate gastric cancer immune status and tumor prognosis. CD163 in cancer cells is involved in the progression and can be upregulated by STAT3. This study replenishes the knowledge of CD163, and further prospective research may allow us to validate whether CD163 is a novel therapeutic target for cancer.

## MATERIALS AND METHODS

### TCGA data retrieval and bioinformatics analysis

Normalized mRNA expression data (RNASeqV2) and patient clinical information of gastric cancer in TCGA database [47] (https://cancergenome.nih.gov/) were downloaded with TCGA Assembler software following the previous study [7]. Spearman correlation analysis was analyzed with R software, and the graphs were mapped with Graphpad Prism software. The overlap genes that correlated with CD163 in gastric cancer tissues and cell lines were firstly analyzed with Venny software, then heatmap of top 20 genes were generated by R software. Protein-Protein interaction network was analyzed with STRING software, and the cellular components or molecular function enrichment analysis of interacted genes were analyzed with Cytoscape software. To predict the potential TF binding site of CD163 promoter, sequence is downloaded from NCBI Gene database (Supplementary Materials) and analyzed with JASPAR software.

### Patient samples and nude mice

All clinical samples including 10 para-cancer tissues, 139 gastric cancer tissues and 10 metastatic lymph nodes were collected from patients in the First Affiliated Hospital of China Medical University in the 2007-2009 period. Tumors had been verified by pathology and the detailed clinical information could be seen in Table 2. All patients in this study were informed and signed a patient informed consent. This study was approved by the Ethics Committee of China Medical University. Female BABL/c nude mice, at 5-6 weeks of age, were purchased from Vital River Laboratory Animal Technology Co.Ltd (Beijing, China).

### Immunohistochemistry

The tissues sections were deparaffinized, rehydrated, antigen retrieval and treated with 3% hydrogen peroxide. After blocking with normal goat serum, sections were incubated with CD163 monoclonal antibody (1:400 diluted with PBS, Abcam, ab189915) at 4°C overnight. Then sections were incubated with Elivision^TM^ plus kit (Maxin, kit-9922) (human clinical samples) or UltraSensitive^TM^ SP kit (Maxin, kit9706) (mice xenograft samples), and detected with DAB kit (Maxin, DAB-0031). All slides were evaluated independently by two investigators in a blinded manner. The number of CD163 positive macrophages were measured with Image J software and classify into 4 grades according to cell number (1: 0-50, 2: 51-100, 3, 101-200, 4, >201 or cell clusters), and CD163 expression in cancer cells were classified into 2 grades (0: negative or weak, 1: moderate or strong). When performing survival analysis, CD163 positive macrophages were further sorted into low expression group (grade 1 and grade 2) and high expression group (grade 3 and grade 4); CD163 in cancer cells also sorted into low expression group (negative) and high expression group (positive).

### Cell culture and transfection

5 gastric cancer cell lines MGC-803, BGC-823, SGC-7901, MKN1 and MKN45 were purchased from the Cell Bank of Chinese Academy of Sciences (Shanghai, China), and cultured in RPMI-1640 containing 10% fetal bovine serum (MKN45 with 20% FBS). To generate CD163 knocking-down BGC-823 and SGC-7901 cell lines, 1×10^4^ cells were seeded into 12-wells plates, and infected with Lentivirus coated shRNA plasmids (5′- GGCTGTGGAGAGGCCATTAAT -3′) or control plasmids (5′-CAGTACTTTTGTGTAGTACAA -3′) according to the instruction (GenePharma, China). Puromycin was added 72 hours after infection at the concentration of 2 μg/mL (Sigma, P9620) until no dying cells were visible. For transfection assays, 2×10^5^ cells were seeded into 6-wells and transfected with Higene (Applygen, C1506) following the manufacture's guidelines. Cells were washed with PBS and harvested with RIPA lysis buffer 48 hour after transfection.

### Western blot

Cells were lysed with RIPA lysis buffer containing protease inhibitor (Roche, 04693124001) and cell supernatant were collected after centrifuging in 4°C. Then 30 μg protein were denaturated and separated by SDS-PAGE. Protein in gels was transferred to a PVDF membraned (Millipore, K5HA3225R), followed blocking with 5 % skimmed milk, incubated with primary antibody overnight and incubated with secondary antibody for 1 hour at room temperature. Finally, membrane were detected with enhanced chemiluminescence reagent (Thermo Fisher Scientific, PK210376). The following antibodies were used: rabbit anti-CD163 monoclonal antibody (1;1000, Abcam, ab189915); mouse anti-flag monoclonal antibody (1:5000, Abmart, 224084); mouse anti-GAPDH monoclonal antibody (1:5000, Kangcheng, kc-5G4).

### Tumor bearing nude mice

In order to obtain xenografts from different tissues, 1×10^6^ SGC-7901 or MKN-45 cells were injected into nude mice by subcutaneous, intraperitoneal and tail vein injection respectively. After 4 weeks, mice were executed and xenografts were peeled out and made into paraffin specimens. To examine the effect of CD163 on tumor proliferation, 1×10^6^ CD163-KD and Control cells were injected into the bilateral axillary. Then xenografts from nude mice were obtained and weighed with an electronic balance, and Paired-samples *t* test was used.

### MTT assay

Cells were digested with 0.25 % trypsin and resuspended with complete medium. Then 2×10^3^ CD163-KD or control BGC-823/SGC-7901 cells were seeded into 96-wells. Next day cells were incubated with 10 μL MTT (10 mg/mL) at 37°C for 4 hours. Subsequently, medium was discarded and 200 μL DMSO was added into wells. After 20 min, the absorbance was measured at 490 nm with a microplate reader. Cell viability was measured for 6 days, and medium was refreshed every two days. All the experiments were repeated three times in triplicates.

### Chromatin immunoprecipitation (CHIP) assay

Logarithmically growing SGC-7901 were seeded in the 10 cm plates (1×10^6^), then transfected with 15μg empty plasmid or flag-STAT3 plasmid. After 48 h, cells were fixed with 1% final concentration formaldehyde solution, followed by sonicating with ultrasonic cell crusher on ice. Cells supernatants were incubated with anti-Flag antibody, and immunoprecipitation with Protein-A beads (Merck Millipore, USA). Subsequently, beads were washed with buffer and eluted with elution buffer. Then cross-links were reversed at 65°C overnight. The DNA was purified with DNA isolation kit (Axygen, USA) and eluted with TE buffer (10mM Tris-HCl, 1mM EDTA, PH=8.0). PCR was conducted using the following primers: forward, 5′- TGAGTTGACTCCGCCTCCAT-3′, reverse, 5′- TCCACTCCTTACTCTCCTGATGC -3′.

### Luciferase reporter assay

Primers used for pGL3 enhancer luciferase report vector were as the following: full length CD163 promoter (PGL3-FULL) (−1000bp to +54 bp), forward, 5′- CTGGTACCTGGGTTCTAGTGAATGTCTCTCTG -3′, reverse, 5′- TCAAGCTTCGCTTTTACCAGCAGATCCAGAGT -3′; deletion type CD163 promoter (pGL3-DEL) (−1000bp to −100bp), forward, 5′- CTGGTACCTGGGTTCTAGTGAATGTCTCTCTG -3′, reverse, 5′- TCAAGCTTATGGAGGCGGAGTCAACTCA -3′. 1×10^5^ cells were seeded into 24-well plate, then 200 ng luciferase report vector (FULL/DEL), 500 ng STAT3-flag or empty vector plasmid were co-transfected with Higene reagent (Applygen, China). 6 h later, medium was replaced with fresh medium containing IL-6 (10 ng/ml, Peprotech, USA) and cultured for another 36 h. Subsequently, cells were lysed and the luciferase activity was measured using Dual Luciferase Reporter Assay System (Promega, USA). All experiments were conducted three times in triplicate.

### Statistics

Independent samples t-test was used to compare the mRNA expression in para-cancer and cancer tissues. One-way ANOVA was use to assess the association between CD163 mRNA expression and clinicalpathologic characters. Line correlation analysis was performed to examine the relationship of CD163 mRNA with other indexes. Chi-Square test was used to analyze the correlation of CD163 positive TAM or cancer cells with clinicalpathologic characters, and Kaplan-Meier was adopted for survival analysis. Paired sample t-test was used to assess the difference between CD163-KD and control cells, and data was presented as Means ± standard deviation (SD). SPSS 19.0 was applied to statistical analysis, and a two-tailed *P* value of < 0.05 indicated statistically significant.

## SUPPLEMENTARY FIGURE AND TABLES



## Abbreviations

TLR2: Toll like receptor 2
NADPH: Nicotinamide adenine dinucleotide phosphate
TCGA: The cancer genome atlas
MTT: 3-(4,5-dimethyl-2-thiazolyl)-2,5-diphenyl-2-H-tetrazolium bromide
STAT3: Signal transducer and activator of transcription 3

## References

[1] Law SK, Micklem KJ, Shaw JM, Zhang XP, Dong Y, Willis AC, Mason DY (1993). A new macrophage differentiation antigen which is a member of the scavenger receptor superfamily. Eur J Immunol.

[2] Schaer DJ, Schaer CA, Buehler PW, Boykins RA, Schoedon G, Alayash AI, Schaffner A (2006). CD163 is the macrophage scavenger receptor for native and chemically modified hemoglobins in the absence of haptoglobin. Blood.

[3] Tarin C, Carril M, Martin-Ventura JL, Markuerkiaga I, Padro D, Llamas-Granda P, Moreno JA, Garcia I, Genicio N, Plaza-Garcia S, Blanco-Colio LM, Penades S, Egido J (2015). Targeted gold-coated iron oxide nanoparticles for CD163 detection in atherosclerosis by MRI. Sci Rep.

[4] Karrasch T, Brunnler T, Hamer OW, Schmid K, Voelk M, Herfarth H, Buechler C (2015). Soluble CD163 is increased in patients with acute pancreatitis independent of disease severity. Exp Mol Pathol.

[5] Rojo-Martinez G, Maymo-Masip E, Rodriguez MM, Solano E, Goday A, Soriguer F, Valdes S, Chaves FJ, Delgado E, Colomo N, Hernandez P, Vendrell J, Chacon MR (2014). Serum sCD163 levels are associated with type 2 diabetes mellitus and are influenced by coffee and wine consumption: results of the Di@bet.es study. PLoS One.

[6] Maniecki MB, Etzerodt A, Ulhoi BP, Steiniche T, Borre M, Dyrskjot L, Orntoft TF, Moestrup SK, Moller HJ (2012). Tumor-promoting macrophages induce the expression of the macrophage-specific receptor CD163 in malignant cells. Int J Cancer.

[7] Taniyama D, Taniyama K, Kuraoka K, Zaitsu J, Saito A, Nakatsuka H, Sakamoto N, Sentani K, Oue N, Yasui W (2017). Long-term follow-up study of gastric adenoma; tumor-associated macrophages are associated to carcinoma development in gastric adenoma. Gastric Cancer.

[8] Etzerodt A, Rasmussen MR, Svendsen P, Chalaris A, Schwarz J, Galea I, Moller HJ, Moestrup SK (2014). Structural basis for inflammation-driven shedding of CD163 ectodomain and tumor necrosis factor-alpha in macrophages. J Biol Chem.

[9] No JH, Moon JM, Kim K, Kim YB (2013). Prognostic significance of serum soluble CD163 level in patients with epithelial ovarian cancer. Gynecol Obstet Invest.

[10] Jensen TO, Schmidt H, Moller HJ, Hoyer M, Maniecki MB, Sjoegren P, Christensen IJ, Steiniche T (2009). Macrophage markers in serum and tumor have prognostic impact in American Joint Committee on Cancer stage I/II melanoma. J Clin Oncol.

[11] Michalkiewicz J, Helmin-Basa A, Grzywa R, Czerwionka-Szaflarska M, Szaflarska-Poplawska A, Mierzwa G, Marszalek A, Bodnar M, Nowak M, Dzierzanowska-Fangrat K (2015). Innate immunity components and cytokines in gastric mucosa in children with Helicobacter pylori infection. Mediators Inflamm.

[12] Hughes PE, Caenepeel S, Wu LC (2016). Targeted therapy and checkpoint immunotherapy combinations for the treatment of cancer. Trends Immunol.

[13] Swart M, Verbrugge I, Beltman JB (2016). Combination approaches with immune-checkpoint blockade in cancer therapy. Front Oncol.

[14] Tamura T, Ohira M, Tanaka H, Muguruma K, Toyokawa T, Kubo N, Sakurai K, Amano R, Kimura K, Shibutani M, Maeda K, Hirakawa K (2015). Programmed death-1 ligand-1 (PDL1) expression is associated with the prognosis of patients with stage II/III gastric cancer. Anticancer Res.

[15] Killock D (2017). Immunotherapy: macrophages hijack anti-PD-1 therapy. Nat Rev Clin Oncol.

[16] Komohara Y, Takeya M (2017). CAFs and TAMs: maestros of the tumour microenvironment. J Pathol.

[17] Malek E, de Lima M, Letterio JJ, Kim BG, Finke JH, Driscoll JJ, Giralt SA (2016). Myeloid-derived suppressor cells: the green light for myeloma immune escape. Blood Rev.

[18] Park JY, Sung JY, Lee J, Park YK, Kim YW, Kim GY, Won KY, Lim SJ (2016). Polarized CD163+ tumor-associated macrophages are associated with increased angiogenesis and CXCL12 expression in gastric cancer. Clin Res Hepatol Gastroenterol.

[19] Dehne N, Mora J, Namgaladze D, Weigert A, Brune B (2017). Cancer cell and macrophage cross-talk in the tumor microenvironment. Curr Opin Pharmacol.

[20] Uhlen M, Fagerberg L, Hallstrom BM, Lindskog C, Oksvold P, Mardinoglu A, Sivertsson A, Kampf C, Sjostedt E, Asplund A, Olsson I, Edlund K, Lundberg E (2015). Proteomics. Tissue-based map of the human proteome. Science.

[21] Yin X, Zhou J, Jie C, Xing D, Zhang Y (2004). Anticancer activity and mechanism of Scutellaria barbata extract on human lung cancer cell line A549. Life Sci.

[22] Brueggmann D, Templeman C, Starzinski-Powitz A, Rao NP, Gayther SA, Lawrenson K (2014). Novel three-dimensional in vitro models of ovarian endometriosis. J Ovarian Res.

[23] Woodfield GW, Chen Y, Bair TB, Domann FE, Weigel RJ (2010). Identification of primary gene targets of TFAP2C in hormone responsive breast carcinoma cells. Genes, Chromosomes Cancer.

[24] Attridge K, Kenefeck R, Wardzinski L, Qureshi OS, Wang CJ, Manzotti C, Okkenhaug K, Walker LS (2014). IL-21 promotes CD4 T cell responses by phosphatidylinositol 3-kinase-dependent upregulation of CD86 on B cells. J Immunol.

[25] Mills CD (2012). M1 and M2 macrophages: oracles of health and disease. Crit Rev Immunol.

[26] Stix G (2007). A malignant flame. Understanding chronic inflammation, which contributes to heart disease, Alzheimer's and a variety of other ailments, may be a key to unlocking the mysteries of cancer. Sci Am.

[27] von Piekartz H, Pudelko A, Danzeisen M, Hall T, Ballenberger N (2016). Do subjects with acute/subacute temporomandibular disorder have associated cervical impairments: a cross-sectional study. Man Ther.

[28] Magana-Maldonado R, Chavez-Cortez EG, Olascoaga-Arellano NK, Lopez-Mejia M, Maldonado-Leal FM, Sotelo J, Pineda B (2016). Immunological evasion in glioblastoma. Biomed Res Int.

[29] He KF, Zhang L, Huang CF, Ma SR, Wang YF, Wang WM, Zhao ZL, Liu B, Zhao YF, Zhang WF, Sun ZJ (2014). CD163+ tumor-associated macrophages correlated with poor prognosis and cancer stem cells in oral squamous cell carcinoma. Biomed Res Int.

[30] Kanno H, Nishihara H, Wang L, Yuzawa S, Kobayashi H, Tsuda M, Kimura T, Tanino M, Terasaka S, Tanaka S (2013). Expression of CD163 prevents apoptosis through the production of granulocyte colony-stimulating factor in meningioma. Neuro Oncol.

[31] Loforte A, Montalto A, Ranocchi F, Della Monica PL, Casali G, Lappa A, Menichetti A, Contento C, Musumeci F (2012). Peripheral extracorporeal membrane oxygenation system as salvage treatment of patients with refractory cardiogenic shock: preliminary outcome evaluation. Artif Organs.

[32] Shabo I, Olsson H, Sun XF, Svanvik J (2009). Expression of the macrophage antigen CD163 in rectal cancer cells is associated with early local recurrence and reduced survival time. Int J Cancer.

[33] Andersen MN, Abildgaard N, Maniecki MB, Moller HJ, Andersen NF (2014). Monocyte/macrophage-derived soluble CD163: a novel biomarker in multiple myeloma. Eur J Haematol.

[34] De Palma M (2016). Origins of brain tumor macrophages. Cancer Cell.

[35] Yang Z, Xie H, He D, Li L (2016). Infiltrating macrophages increase RCC epithelial mesenchymal transition (EMT) and stem cell-like populations via AKT and mTOR signaling. Oncotarget.

[36] Berraondo P, Minute L, Ajona D, Corrales L, Melero I, Pio R (2016). Innate immune mediators in cancer: between defense and resistance. Immunol Rev.

[37] Allavena P, Sica A, Garlanda C, Mantovani A (2008). The Yin-Yang of tumor-associated macrophages in neoplastic progression and immune surveillance. Immunol Rev.

[38] Hasita H, Komohara Y, Okabe H, Masuda T, Ohnishi K, Lei XF, Beppu T, Baba H, Takeya M (2010). Significance of alternatively activated macrophages in patients with intrahepatic cholangiocarcinoma. Cancer Sci.

[39] Komohara Y, Takeya M (2016). CAFs and TAMs: maestros of the tumour microenvironment. J Pathol.

[40] Bover LC, Cardo-Vila M, Kuniyasu A, Sun J, Rangel R, Takeya M, Aggarwal BB, Arap W, Pasqualini R (2007). A previously unrecognized protein-protein interaction between TWEAK and CD163: potential biological implications. J Immunol.

[41] Michaelson JS, Amatucci A, Kelly R, Su L, Garber E, Day ES, Berquist L, Cho S, Li Y, Parr M, Wille L, Schneider P, Wortham K (2011). Development of an Fn14 agonistic antibody as an anti-tumor agent. MAbs.

[42] Di Martino L, Dave M, Menghini P, Xin W, Arseneau KO, Pizarro TT, Cominelli F (2016). Protective role for TWEAK/Fn14 in regulating acute intestinal inflammation and colitis-associated tumorigenesis. Cancer Res.

[43] Siveen KS, Sikka S, Surana R, Dai X, Zhang J, Kumar AP, Tan BK, Sethi G, Bishayee A (2014). Targeting the STAT3 signaling pathway in cancer: role of synthetic and natural inhibitors. Biochim Biophys Acta.

[44] Chai EZ, Shanmugam MK, Arfuso F, Dharmarajan A, Wang C, Kumar AP, Samy RP, Lim LH, Wang L, Goh BC, Ahn KS, Hui KM, Sethi G (2016). Targeting transcription factor STAT3 for cancer prevention and therapy. Pharmacol Ther.

[45] Tye H, Kennedy CL, Najdovska M, McLeod L, McCormack W, Hughes N, Dev A, Sievert W, Ooi CH, Ishikawa TO, Oshima H, Bhathal PS, Parker AE (2012). STAT3-driven upregulation of TLR2 promotes gastric tumorigenesis independent of tumor inflammation. Cancer Cell.

[46] Xu YY, Guo M, Yang LQ, Zhou F, Yu C, Wang A, Pang TH, Wu HY, Zou XP, Zhang WJ, Wang L, Xu GF, Huang Q (2017). Regulation of CD44v6 expression in gastric carcinoma by the IL-6/STAT3 signaling pathway and its clinical significance. Oncotarget.

[47] Schmidt RC, Bart HL, Nyingi DW, Gichuki NN (2014). Phylogeny of suckermouth catfishes (Mochokidae: Chiloglanis) from Kenya: the utility of Growth Hormone introns in species level phylogenies. Mol Phylogene Evol.

