# Supplementary material for: CD163 as a novel target gene of STAT3 is a potential therapeutic target for gastric cancer
Source: Oncotarget. 2017 Aug 14;8(50):87244–62. doi: 10.18632/oncotarget.20244 (PMC5675630; doi:10.18632/oncotarget.20244)
Supplement: Supplementary file 1 [file oncotarget-08-87244-s001.pdf]

CD163 as a novel target gene of STAT3 is a potential therapeutic target for gastric cancer

SUPPLEMENTARY MATERIALS

Sequence of CD163 promoter (-1000 to +100) (0 to 100 is marked with red color)

TGGGTTCTAGTGAATGTCTCTCTGGAAGGCTGGAGTTGCTCTTTAATTCCCCATTTCAGGCCCATCACTAACACCTGACA

ATATGTATGCCATGATGAACTATTAGTAGTTCTTTTGAGCTTATATGTTTTTATATACAAATATTCATTTGTATGTGCTA

TTTTTTATGCCTTAAACTCCTTTATCATTTGTCCCTTTGGCAATGTTTTTCTCATCTTTTAGGACACAATTTAAGCCTGT

CCTCAGAGAAAATAGTTTTCTGACTGTTCATTCCTTTCTCTCAATAGATAGGACTACTTTGTCCATTATTTTTAACACTG

CACTTTAGCTTTATGTTCATTGTTGTTATCATATGTGTTTCTCTTACTTGAATCTGAGTTATATAGAGCTAAAATCATGT

GTTGCTAATTTTTGTTTCACCATTTGTAATATCAGTAATAGTCATGGCTAATTCTCTTGGTGTACTTCATCCCATTAGAA

AAGAAATGACAAATGCTGTGTCTCAACAACTTACACAAAATTACTCATTAGACACATTTGATTATGGAAATAAAATTAAA

AGTGCATATGATAAAATGTTATTTAATTATGTTTTGCCTGTTTTGCTTTAGTTTTTTACATAATTTTTCTACATGACAAT

TAGTAATTTTTTGTGTCTTATATATTTGTCCAAAATGAAGTTCAAAAATGTAAAATATTTAATTCAGCAACAGCAGCATA

TGAGTTAGTATTTCCTCTAATTTTTCGAAATCTGTGGGAAGTGTTTCCCAATTTCCTTTGGTTGTTTCATGTGCTATATT

GAAGAAAACATGAGTATGAAATGGAACCTCAGCTCTTTCAATGACTTCCCTTTTTGAGTTGACTCCGCCTCCATATGTAG

CCTTTTCATTTTCATGAAAGTGAAGTGATTTTTAGAATTCTTAGTTGTTTTCTTTAGAAGAACATTTCTAGGGAATAATA

CAAGAAGATTTAGGAATCATTGAAGTTATAAATCTTTGGAATGAGCAAACTCAGAATGGTGCTACTTGAAGACTCTGGAT

CTGCTGGTAAAAGCTTCTCATTTATTCTACATTTCCCCTTTAATGGGGTATGTAATTATT

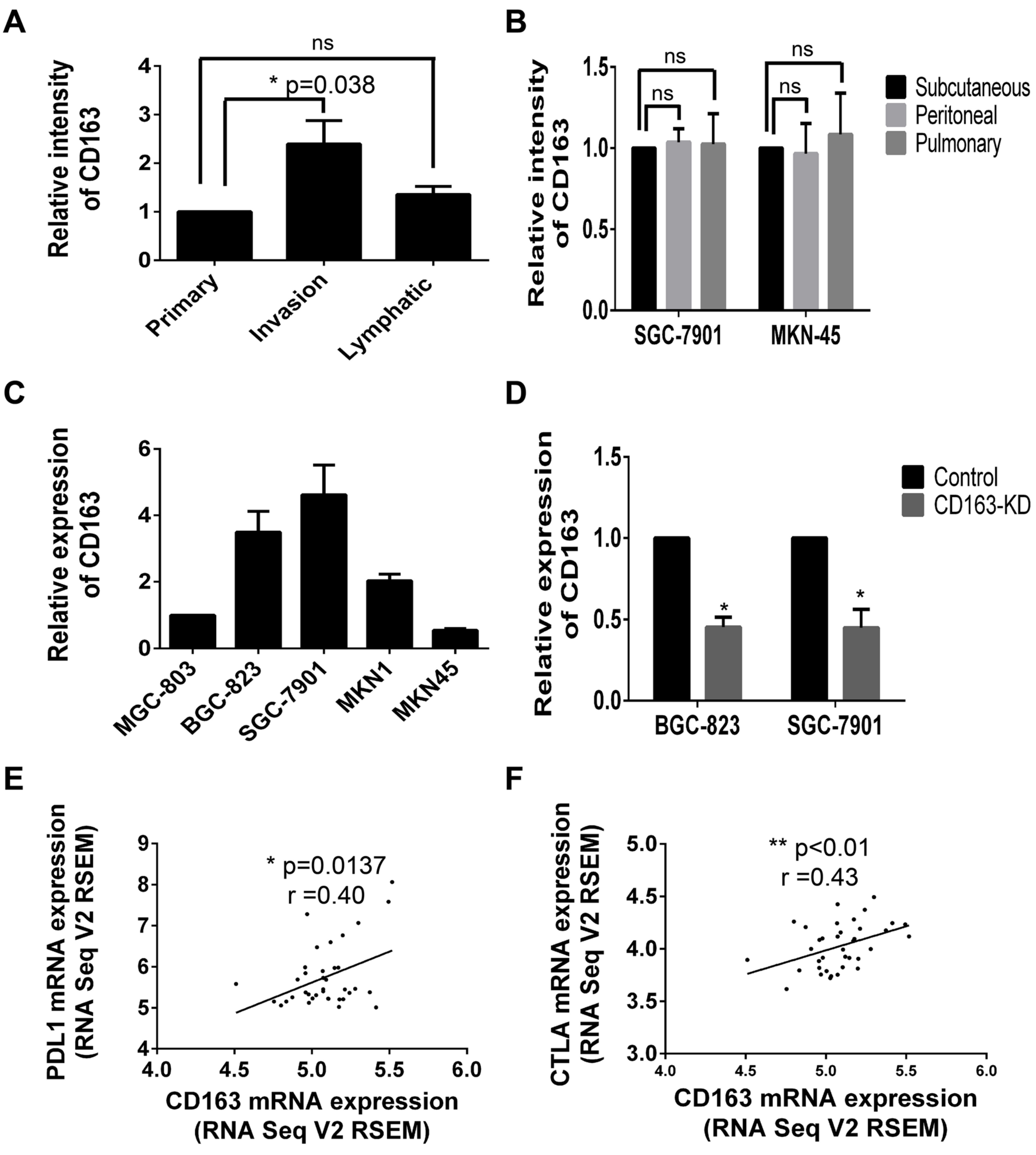

Supplementary Figure 1: (A), Relative expression of CD163 in gastric mucosa (primary), muscle layer (invasion) and metastatic lymph node (lymphatic) is analyzed with Image J. **(B)**, Relative CD163 expression in different tissues xenografts (subcutaneous, peritoneal and pulmonary cancer) from SGC-7901 and MKN-45 is analyzed with Image J. **(C)** Relative CD163 expression in gastric cancer cell lines is analyzed with Image J. **(D)**, Relative CD163 expression in CD163-KD or Control stable cell lines is analyzed with Image J. **(E, F)**, Correlationship between CD163 and PD-L1, CTLA4 in 37 gastric cancer cell lines are analyzed by R software.

Supplementary Table 1: Top 10 functional pathways of CD163 co-expressing genes

Pathway ID	Pathway name	Gene number	P value
GO.0002376	immune system process	70	1.62E-29
GO.0006952	defense response	61	1.62E-29
GO.0006955	immune response	59	2.86E-29
GO.0050776	regulation of immune response	43	9.30E-23
GO.0002684	positive regulation of immune system process	42	1.65E-21
GO.0002253	activation of immune response	32	1.89E-20
GO.0006954	inflammatory response	32	1.89E-20
GO.0050778	positive regulation of immune response	35	2.43E-20
GO.0002682	regulation of immune system process	49	1.23E-19
GO.0045087	innate immune response	42	1.23E-19

Supplementary Table 2: COX regression analysis of the association of clinicopathological characteristics and CD163 expression in TAMs with gastric cancer survival

Parameters	Univariate analysis		Multivariate analysis	
	Hazard ratio (95%CI)	*P*	Hazard ratio (95%CI)	*P*
**Age (y)**				
<60 *vs.* ≥60	1.125(0.733-1.726)	0.591		
**Gender**				
Male *vs.* Female	0.765(0.476-1.229)	0.268		
**Histologic grade**				
Well & Moderate *vs.* Poor	1.549(0.994-2.414)	0.053		
**Depth of invasion**	``		`	
T1 *vs.* T2-T4	4.179(2.153-8.111)	**< 0.001**	3.249(1.592-6.631)	**0.001**
**Lymph node status (pN)**				
N0 *vs.* N1-N3	2.721(1.760-4.207)	**< 0.001**	2.363(1.107-5.044)	**0.026**
**Pathological stage (TNM)**				
I-II *vs.* III-IV	2.787(1.805-4.303)	**< 0.001**	1.030(0.471-2.253)	0.941
**CD163 in TAMs**				
Low *vs.* High	1.861(1.194-2.902)	**0.006**	1.718(1.093-2.700)	**0.019**

NOTE: Bold, significant values < 0.05.

Supplementary Table 3: COX regression analysis of the association of clinicopathological characteristics and CD163 expression in cancer cells with gastric cancer survival

Parameters	Univariate analysis		Multivariate analysis	
	**Hazard ratio (95%CI)**	***P***	**Hazard ratio (95%CI)**	***P***
**Age (y)**				
<60 *vs.* ≥60	1.125(0.733-1.726)	0.591		
**Gender**				
Male *vs.* Female	0.765(0.476-1.229)	0.268		
**Histologic grade**				
Well & Moderate *vs.* Poor	1.549(0.994-2.414)	0.053		
**Depth of invasion**	``		`	
T1 *vs.* T2-T4	4.179(2.153-8.111)	**< 0.001**	3.287(1.600-6.754)	**0.001**
**Lymph node status (pN)**				
N0 *vs.* N1-N3	2.721(1.760-4.207)	**< 0.001**	2.235(1.074-4.654)	**0.032**
**Pathological stage (TNM)**				
I-II *vs.* III-IV	2.787(1.805-4.303)	**< 0.001**	1.067(0.497-2.289)	0.868
**CD163 in Cancer cells**				
Low *vs.* High	1.781(1.153-2.751)	**0.009**	1.595(1.028-2.473)	**0.037**

NOTE: Bold, significant values < 0.05
